# Primary pulmonary leiomyosarcoma with coexistent pulmonary aspergillosis: a case report

**DOI:** 10.11604/pamj.2022.42.135.34116

**Published:** 2022-06-20

**Authors:** Ahmed Badheeb, Nasser Al Gharem, Shehab Al Hammadi, Said Elsagheer, Mohamed Badheeb, Faisal Ahmed

**Affiliations:** 1 Department Oncology, King Khalid Hospital, Najran, Saudi Arabia,; 2 Department of Internal Medicine, Faculty of Medicine, Hadhramout University, Hadhramout, Yemen,; 3 Department of Radiology, King Khalid Hospital, Najran, Saudi Arabia,; 4 Department of Pathology, King Khalid Hospital, Najran, Saudi Arabia,; 5 Department of Thoracic Surgery, King Khalid Hospital, Najran, Saudi Arabia,; 6 Department of General Medicine, King Khalid Hospital, Najran, Saudi Arabia,; 7 Urology Research Center, Al-Thora General Hospital, Department of Urology, School of Medicine, Ibb University of Medical Science, Ibb, Yemen

**Keywords:** Primary pulmonary leiomyosarcoma, aspergillosis, chemotherapy, case report

## Abstract

Coexisting primary pulmonary leiomyosarcoma (PPL) with pulmonary Aspergillosis in immunocompetent patients is a rare occurrence. Here, we presented a 54-year-old woman presented with a dry cough for two months. Bronchoscopy revealed pulmonary aspergillosis. The patient was treated with antifungal therapy for one month without improvement. To evaluate further, a chest computed tomography (CT) scan showed a large heterogeneous enhancing mass in the lower lobe of the left lung with left atrium thrombosis. Computed tomography-guided biopsy was performed, and histopathology demonstrated the diagnosis of PPL. The metastasis workup staging showed multiple metastases in vertebrae, scapula, rib, and liver. The patient was treated with chemotherapy followed by tumor bed radiotherapy. Unfortunately, her general condition worsened, and she passed away with overall survival of fourteen months. In conclusion, clinicians should be alert to underlying malignant disease if airway Aspergillus infection is suspicious in patients without strong risk factors for invasive fungal infection.

## Introduction

Leiomyosarcomas are sporadic malignant tumors that frequently arise from soft tissues, smooth muscles of the uterus, or gastrointestinal tract. Primary pulmonary leiomyosarcomas (PPLs) are uncommon, with few reported cases in the literature [1]. Primary pulmonary leiomyosarcomas is a subtype of pulmonary sarcoma, accounting for less than 0.5% of all malignant pulmonary neoplasms. It can originate from the smooth muscles of pulmonary parenchyma, interstitium, or pulmonary vasculatures [1,2]. The clinical presentation ranges from an utterly asymptomatic presentation with accidental discovery on radiologic images to a wide range of nonspecific symptoms such as cough, dyspnea, and chest discomfort [2]. Radiologic imaging modalities help in detecting the tumor´s location and exclude extra-thoracic origin. However, the final diagnosis is established mainly by histological examination of specimens [3,4]. Aspergillosis is primarily seen in immunocompromised patients or those with underlying chronic lung diseases. Aspergillosis coexisting with PPL in the immunocompetent is extremely rare [5,6]. We present a case of Aspergillosis coexisting with PPL with multiple metastases in 54-year-old women and discuss the current knowledge on the etiology, diagnosis, and treatment of this condition.

## Patient and observation

**Patient information:** a 54-year-old woman presented with a dry cough and chest discomfort for two months. There was no history of fever, anorexia, hemoptysis, dyspnea, or weight loss. Her medical condition and family history were unremarkable, and she had no history of smoking or specific medication. The patient mentioned a history of abdominal hysterectomy for a large benign fibroid tumor in the uterus four years ago.

**Clinical findings:** the patient vital signs were stable (blood pressure: 120/70 mmHg, respiratory rate: 14 respirations per minute, pulse rate: 61 beats per minute). The chest examination revealed decreased breath sounds and fine crackles in the left lung base, but was otherwise unremarkable.

**Figure 1:**
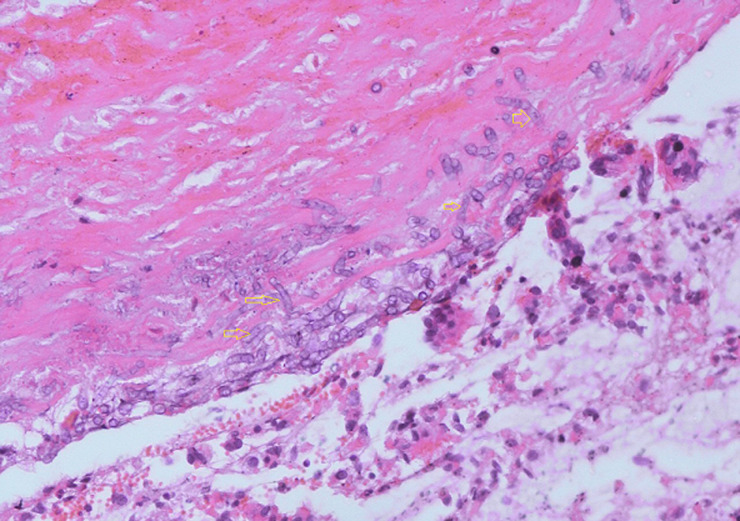
specimen from bronchoscopic biopsy showing numerous hyphae with septation consistent with aspergillosis (arrows)

**Diagnostic assessment:** laboratory tests revealed elevated acute-phase reactants, including erythrocyte sedimentation rate (ESR) and C-reactive protein (CRP). Other laboratory tests were within normal ranges, including basic metabolic panel, renal function tests, and liver function tests. Sputum from the patient was negative for acid-fast bacilli and sputum culture. Initial chest X-ray revealed with well-defined nodular mass in the lower lobe of the left lung. The patient underwent a bronchoscopic evaluation that revealed a mass is invading the left main bronchus. A culture of bronchoalveolar lavage fluid confirmed the presence of Aspergillus fumigatus (Figure 1).

**Therapeutic interventions:** the patient was diagnosed with pulmonary aspergillosis and treated with antifungal therapy (Caspofungin 70 mg on the first day, then 50 mg daily intravenous infusion for one month).

**Figure 2:**
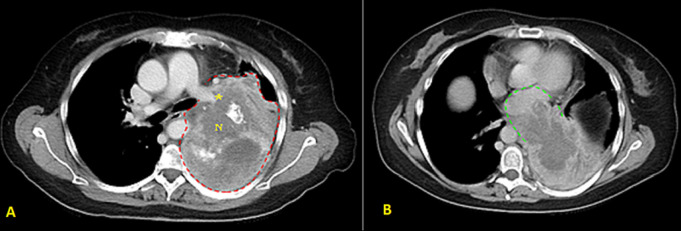
chest computerized tomography scan showing: A) large lung soft tissue mass lesion with areas of necrosis (red dashed line); B) left inferior pulmonary vein and left atrium thrombosis (green dashed line)

**Figure 3:**
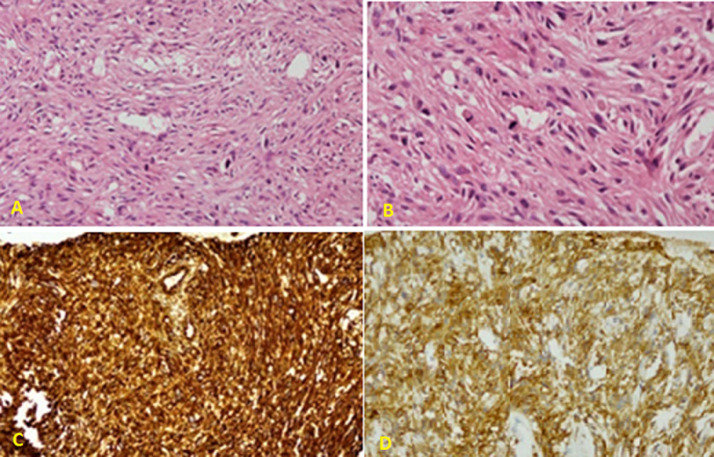
pathologic examination showing: A) proliferating spindle cells forming interlacing bundles and high mitotic figures (HE stain x 200); B) proliferating spindle cells forming interlacing bundles and high mitotic figures (HE stain x 400); C) immunohistochemistry stain showing diffuse positivity of vimentin; D) immunohistochemistry stain showing positive smooth muscle actin

**Figure 4:**
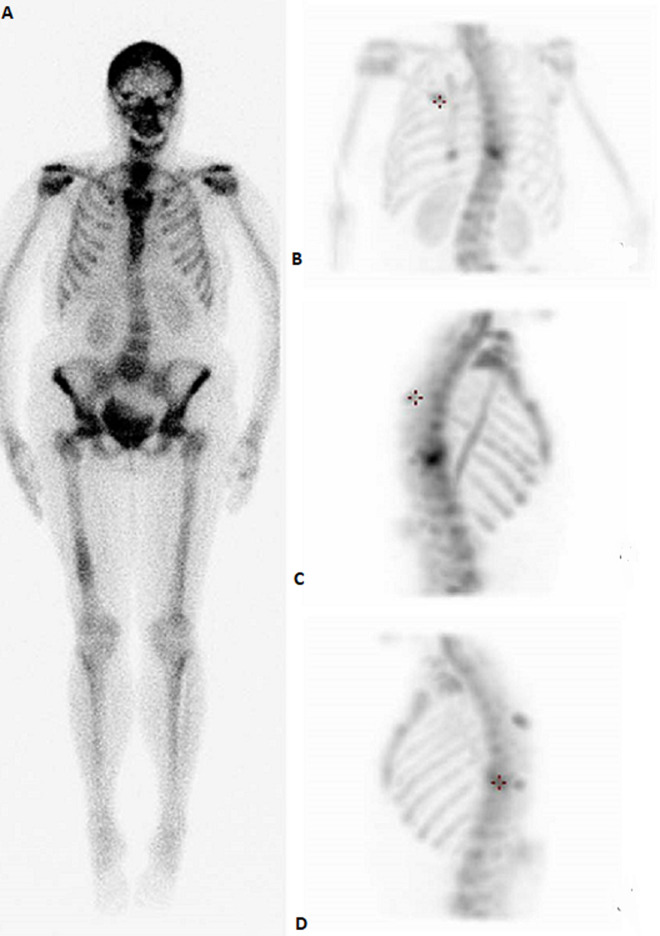
bone scan in anterior view (A), posterior view (B), and chest oblique views (C,D) showing multiple hot spots in the inferior angle of the scapula, 10^th^ left rib posteriorly, and the vertebral discs suggestive of metastasis (stars)

**Figure 5:**
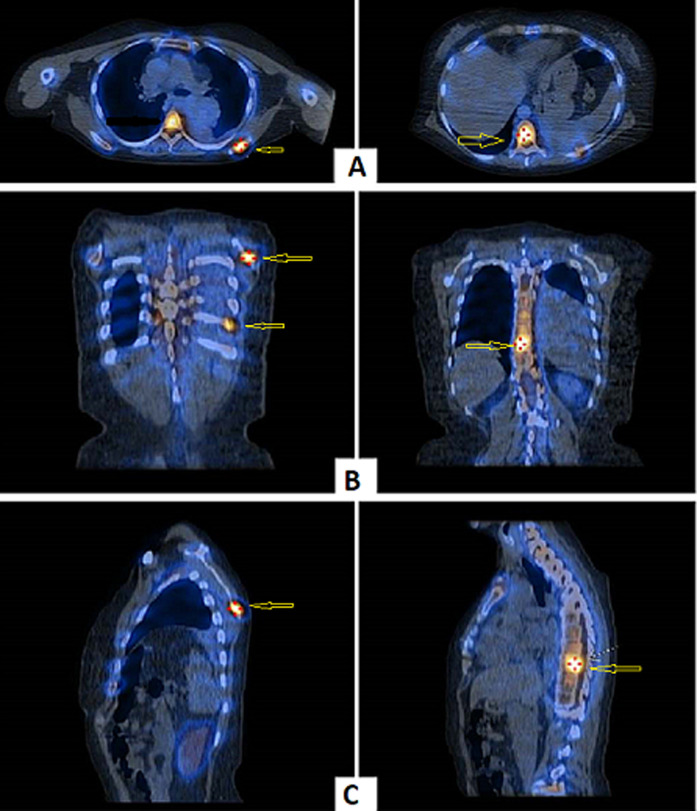
functional single-photon emission computed tomography images in axial view (A), coronal view (B), and sagittal view (C) showing the increased area of activity in the inferior angle of the scapula, 10^th^ left rib posteriorly, and the vertebral discs suggestive of metastasis (arrows)

**Figure 6:**
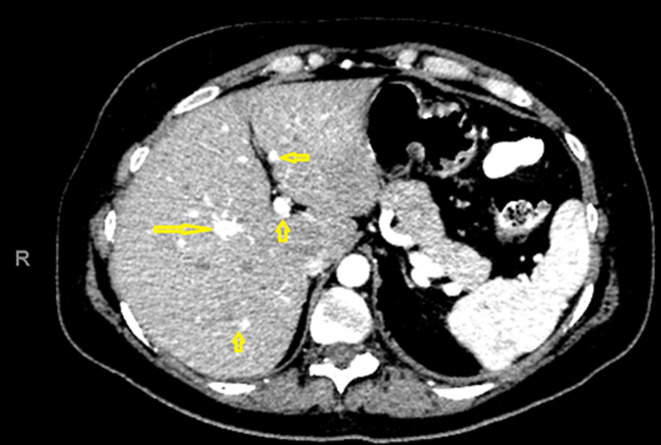
abdominal computerized tomography scan showing hepatic hypodense lesions (arrow)

**Follow-up and outcome:** after one month of Caspofungin administration, she presented with worsening symptoms and was admitted for further evaluation. Chest computed tomography (CT) scan showed an 8 x 4 cm heterogeneous, well-defined left lower lobe mass with areas of soft tissue enhancement and necrosis, invasion of the left atrium, the left main bronchus, and left pulmonary artery, and compression of the left inferior pulmonary vein (Figure 2). There was no lymph node enlargement in the hilum and mediastinum. As the trans-bronchial lung biopsy showed no malignant feature, a CT-guided core needle biopsy of the left lung mass was performed, and the histopathological examination of the specimen demonstrated that the tumor was composed of spindle cells with marked nuclear pleomorphism and high mitotic activity with areas of necrosis and suggestive for PPL. Immunohistochemistry (IHC) stains were positive diffusely for smooth muscle actin (SMA), vimentin, and h-Caldesmon. The tumor was negative for CKAE1/AE3, Ck7, CK5/6, EMA, Calretinin, S100, P63, CD34, BCl2, Desmin, Myogenin, and CD117 immunostains ruling out other possibilities and confirming the diagnosis of leiomyosarcoma (Figure 3). For metastasis workup, Magnetic Resonance Imaging (MRI) of the spine revealed scattered abnormal signal intensity lesions within the bone marrow of multiple vertebrae. The whole-body scan and SPECT (single-photon emission computed tomography) scan revealed an increased radiotracer activity in the inferior angle of the scapula, 10^th^ left rib posteriorly, and the vertebral discs (Figure 4, Figure 5). Further, imaging with an abdominal CT scan with intravenous injection revealed multiple small liver lesions suggestive of metastasis (Figure 6).

As surgical removal was not an option. For that, the patient was transferred to the oncology department and received palliative chemotherapy with gemcitabine and docetaxel (gemcitabine 900 mg/m^2^on days 1 and 8, docetaxel 75 mg/m^2^on day 8 only) with a marked clinical response. After three cycles of chemotherapy, the follow-up CT scan showed a partial response according to RECIST (response evaluation criteria in solid tumors) criteria (The active enhancing components and size were decreased. While the intravascular tumor thrombosis remained unchanged) [7]. After eight months of chemotherapy administration, the patient complained of peripheral neuropathy. The CT scan showed disease progression; therefore, she was switched to second-line chemotherapy using doxorubicin (60 mg/m^2^) monotherapy every three weeks. But the follow-up CT scan showed disease progression. Therefore, Dacarbazine (850 mg/m^2^) every three weeks was added for two cycles. During treatment, the patient developed grade 3 neutropenia and could not tolerate further chemotherapy. Subsequently, she underwent single fraction palliative chest radiotherapy of 8 Gy. Unfortunately, the patient´s general condition worsened, and she died two months later, with overall survival of fourteen months from the initial presentation.

**Patient perspective:** during treatment, the patient was satisfied with the level of care provided to her. Early with palliative therapy, she remained socially and functionally active. The patient understood the terminal stage of her illness with tremendous support from her family.

**Informed consent:** the consent was obtained from the patient's family following her death.

## Discussion

Primary pulmonary sarcomas are extremely rare, representing 0.2-1% of lung cancer [1,8]. Primary pulmonary leiomyosarcoma represents 30% of primary pulmonary sarcomas, either intrapulmonary (parenchymal) or present as bronchial masses. The parenchymal form can grow significantly due to its silent nature [8]. Primary pulmonary leiomyosarcoma can arise from the pulmonary smooth muscles, interstitium, and pulmonary vasculature, as in our case [8,9]. Leiomyosarcomas were observed in patients with variant age groups; however, PPLs remain more common in older adults with a higher tendency to affect males, and 90% of these patients have a history of heavy smoking; making our patient age, history, and gender; less prevalent [9]. The symptoms of PPL are variable and nonspecific, depending on the site and size of the tumor. It may be discovered incidentally with radiological imaging; it can also present with chronic cough, shortness of breath, and chest pain [3,10]. Few cases of PPL present with pulmonary aspergillosis have been reported, such as Olobatoke *et al*. and our case [5]. Pulmonary aspergillosis presentation varies based on patients' immune state, with invasive disease observed predominantly in immunocompromised patients or chronic underlying lung diseases [6,11]. The rarity of such presentation and the unremarkable medical history of our patient together made the diagnosis extremely challenging [5].

Biopsy of the tumor, mainly if it is centrally located, frequently reveals only one component, and peripheral tumors are challenging to reach endoscopically [12]. In our case, the bronchoscopy did not show cancer, and the final diagnosis was made after CT guided biopsy. Radiologically, PPLs appear as well-defined smooth nodules, lobular homogenous nodules, or solitary necrotic masses, similar to bronchogenic carcinoma. The absence of lymph node involvement is a distinctive feature that helps in differentiating PPLs from other bronchogenic carcinoma [4]. Histopathological examination of the biopsy specimen is the gold standard for PPL diagnosis [3]. The gross examination may reveal a grey or white firm surface. On microscopic evaluation, malignant spindle cells with cigar-shaped nuclei organized in an interwoven fascicle pattern can be observed. Additionally, mitotic figures, multinucleation, nuclear atypia, marked vascularity, sparse cytoplasm, and necrosis are prevalent. Immunohistochemistry (IHC) staining typically shows positivity to actin, smooth muscle actin (SMA), desmin, and vimentin antibodies. Lack of positivity for soft muscle markers and reactivity of other affirmative markers, such as CD34, S100, or multiple cytokeratins, should raise the suspicion for an alternative diagnosis [3]. In our case, Vimentin, as a mesenchymal marker, was positive and epithelial markers for different types of carcinomas were all negative, and h-Caldesmon, and α-SMA were strongly positive, confirming that the origin of the tumor was smooth muscle. A similar case was reported by Yata *et al*. [13].

Survival was correlated with pathological differentiation, stage, the primary site of the tumor, lymph node involvement, and distant metastases. Nevertheless, tumor size did not affect the outcome [4,10,14]. The outcome for metastatic patients, as with our case, remains poor, with a median reported overall survival of fewer than 18 months [15,16]. Olobatoke *et al*. reported a case of PPL coexisting with Aspergillosis in a 66-year-old man complicated with Acinetobacter pneumonia and succumbed to cardiac arrest [5]. According to Ishida *et al*. five patients had a 9-month median survival time [17]. Davis *et al*. reported a 12-month median survival time in 15 patients with PPL after surgery [18]. Huwer *et al*. examined seven patients with pulmonary carcinosarcoma out of 2,400 lung cancer cases and reported that tumor recurrence or distant metastasis of the sarcoma component was associated with high mortality [12].

Palliative chemotherapy is the primary treatment for metastatic PPL in patients who are not candidates for surgery to achieve systemic control of the illness and improve the quality of life [19]. According to the National Comprehensive Cancer Network (NCCN) guidelines 2021, either combination regimens of chemotherapy can be used in the first line are setting like doxorubicin - dacarbazine, AIM (Doxorubicin-Ifosfamide-Mesna), MAID (doxorubicin-ifosfamide-mesna-dacarbazine), gemcitabine combinations with docetaxel, vinorelbine or dacarbazine, or any single agent like doxorubicin, ifosfamide, mesna, dacarbazine, docetaxel, vinorelbine, dacarbazine, temozolomide, liposomal doxorubicin [20]. Gemcitabine and docetaxel combination is effective in soft-tissue sarcomas, lower hospitalization rate, and has been established as a therapeutic option for advanced uterine leiomyosarcoma [20]. In our patient, the first line was administrated but was not effective. The patient switched to two more lines of chemotherapy, dacarbazine followed by doxorubicin, but she progressed with deterioration of the performance status. Later on, she received one fraction of palliative radiotherapy to control the chest pain. Unfortunately, the patient expired after 14 months from the diagnosis. The current case highlights the importance of a thorough evaluation of pulmonary aspergillosis to exclude lung malignancies, particularly in patients with no history of lung diseases. A high level of suspicion and broadening the differential diagnosis in non-responding patients is crucial for early detection and management [5]. To our knowledge, this is the second reported case of PPL with coexicittong pulmonary aspergillosis. The previous issue was reported by Olobatoke *et al*. [5].

## Conclusion

Primary pulmonary leiomyosarcomas represent an infrequent clinicopathological entity that requires early detection, complete diagnostic workup, and staging. Additionally, clinicians should be alert to underlying malignant disease if airway Aspergillus infection is suspicious in patients without strong risk factors for invasive fungal disease.
